# Supplementary material for: Structural Comparison of Diverse HIV-1 Subtypes using Molecular Modelling and Docking Analyses of Integrase Inhibitors
Source: Viruses. 2020 Aug 26;12(9):936. doi: 10.3390/v12090936 (PMC7552036; doi:10.3390/v12090936)
Supplement: Supplementary file 1 [file viruses-12-00936-s001.pdf]

viruses

Supplementary Tables

Supplementary Table 1. Binding affinities (ΔG in kcal/mol).

Drug	02-AG	CON-C	5U1C	6RWM
Ral	−7.08	−7.37	−6.88	–
Elv	−6.95	−6.83	−6.75	–
Dol	−7.1	−7.3	−7.22	–
Bic	−7.17	−7.69	−7.55	−6.81
Cab	−6.77	−6.48	−6.79	–

Supplementary Table 2. Inhibition constants (Ki in μM).

Drug	02-AG	CON-C	5U1C	6RWM	EC50 (nM)
Ral	6.19	3.79	8.68	–	0.173
Elv	7.71	9.45	10.82	–	0.362
Dol	5.98	4.26	4.88	–	0.08*
Bic	5.31	2.2	2.79	9.77	0.022*
Cab	10.46	17.09	10.11	–	0.56*

*: Susceptibilities of HIV-1 to INSTI in TZM-bl cells [61].

Supplementarty Figures

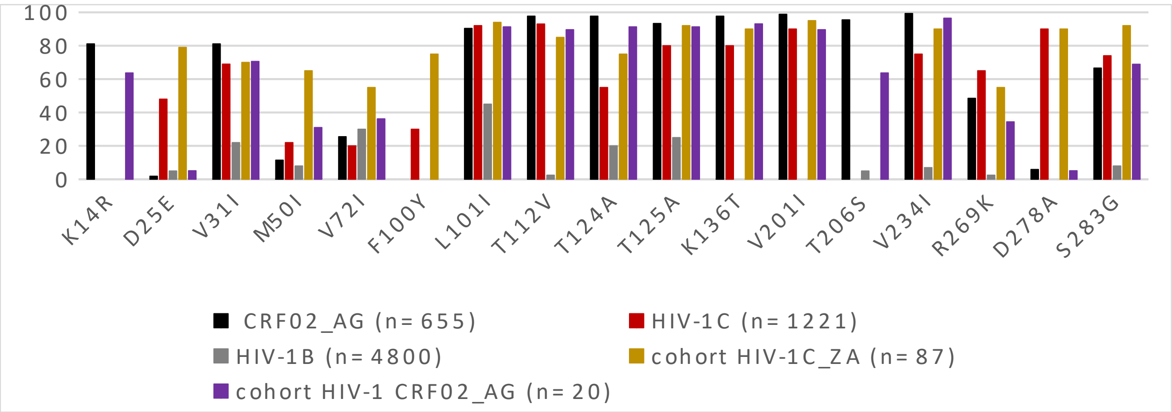

Supplementary Figure S1. HIV-1C and CRF02_AG integrase mutation profiling. Integrase mutation profiling of consensus sequences generated using the database-derived HIV-1CZA sequences HIV-1B (n = 4800), HIV-1C (n = 1221), and CRF02_AG (655). Cohort sequences HIV-1C-ZA (n = 87) and cohort sequences HIV-1 CRF02_AG (n = 37) identified 17 NOPs (D25E, V31I, M50I, V72I, F100Y, L101I, T112V, T124A, T125A, K136Q, I151V, V201I, T218I, V234I, R269K, D278A, S283G) and 13 NOPs (K14R, V31I, V72I, L101I, T112V, T124A, T125A, K136T, I151V, V201I, T206S, V234I, S283G) ,respectively. Among the NOPs, 7 were further increased in our cohort.

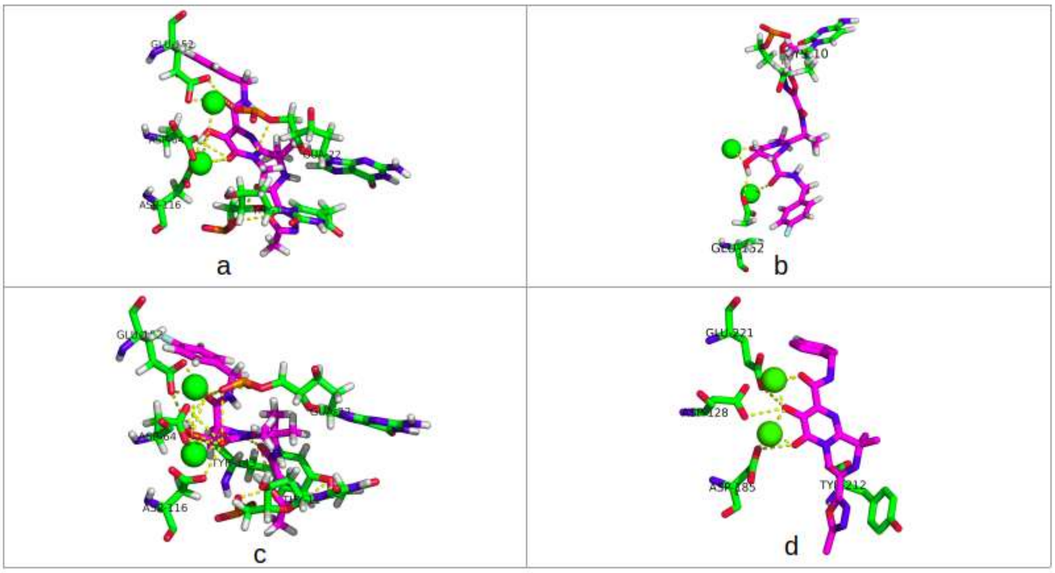

Supplementary Figure S2. Interaction diagram showing different polar contacts being formed between RAL and different IN subtypes. **a)** CRF_O2_AG IN, **(b)** Subtype B IN,**(c)** Subtype C IN, and **(d)** PFV IN. RAL shown in magenta and colored by element, MG ions shown as green spheres, and IN residues in green are labelled.

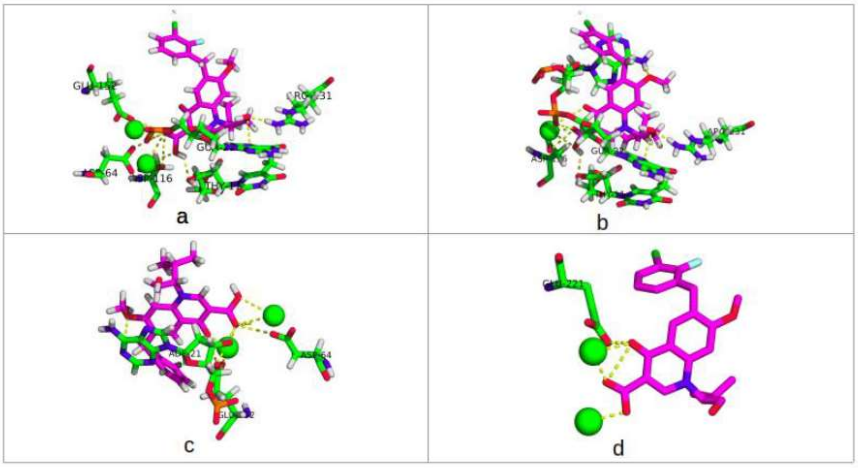

Supplementary Figure S3. Interaction diagram showing different polar contacts being formed between EVG and different IN subtypes. **(a)** CRF_O2_AG IN, **(b)** Subtype B IN, **(c)** Subtype C IN, and **(d)** PFV IN. EVG shown in magenta and colored by element, MG ions shown as green spheres, and IN residues in green are labelled.

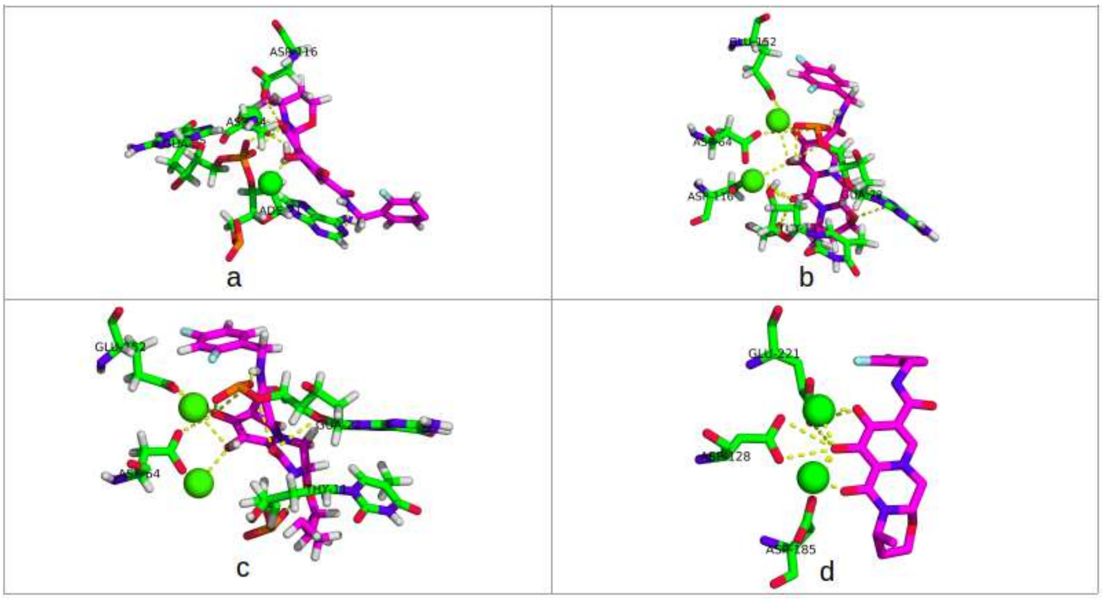

Supplementary Figure S4. Interaction diagram showing different polar contacts being formed between DTG and different IN subtypes. **(a)** CRF_O2_AG IN, **(b)** Subtype B IN, **(c)** Subtype C IN, and **(d)** PFV IN. DTG shown in magenta and colored by element, MG ions shown as green spheres, and IN residues in green are labelled.

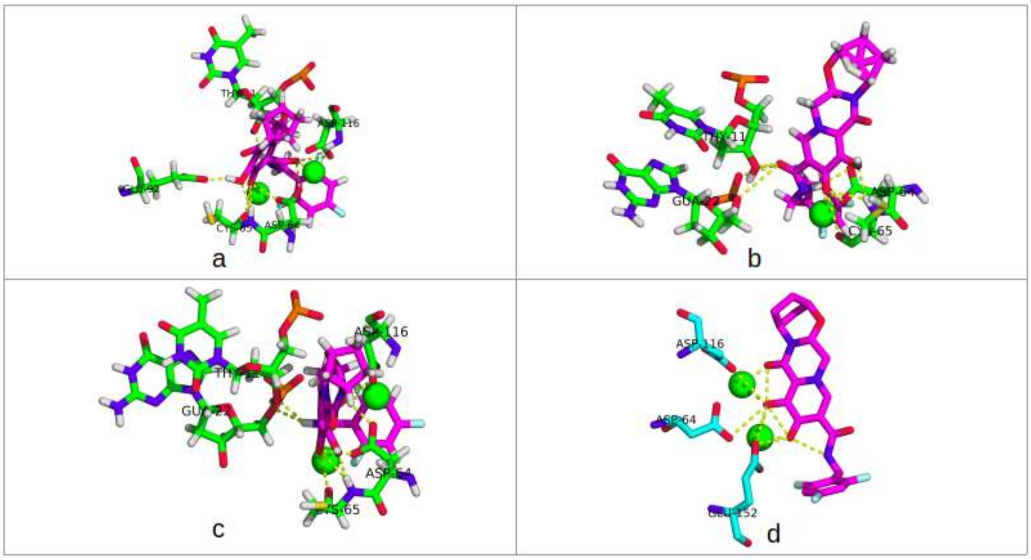

Supplementary Figure S5. Interaction diagram showing different polar contacts being formed between BIC and different IN subtypes. **(a)** CRF_O2_AG IN, **(b)** Subtype B IN, **(c)** Subtype C IN, and **(d)** SIV IN. BIC shown in magenta and colored by element, MG ions shown as green spheres, and IN residues in green are labelled.

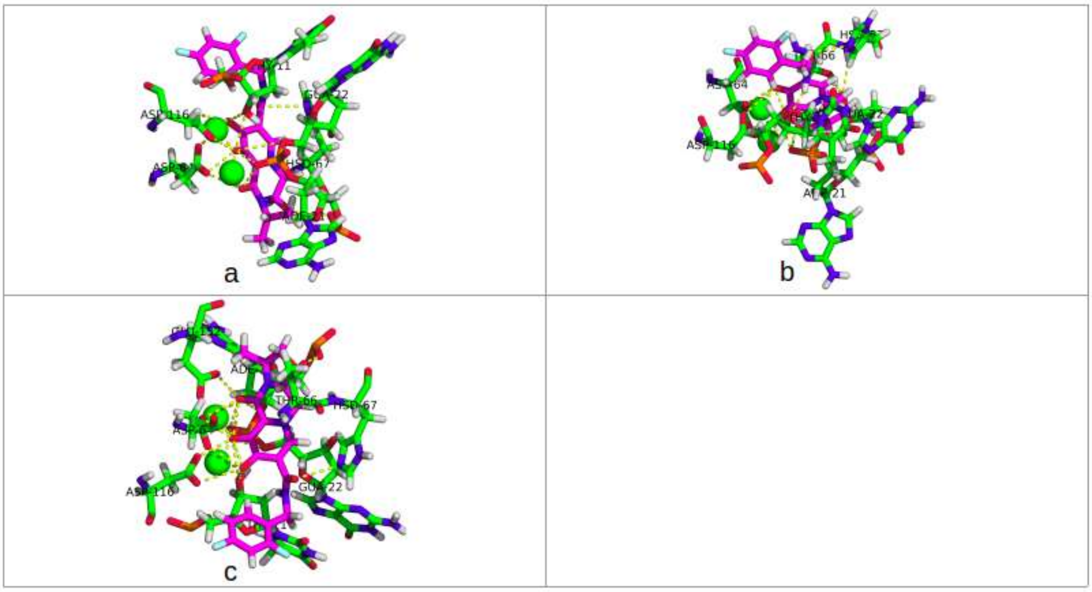

Supplementary Figure S6. Interaction diagram showing different polar contacts being formed between CBT and different IN subtypes. **(a)** CRF_O2_AG IN, **(b)** Subtype B IN, **(c)** Subtype C IN. CBT shown in magenta and colored by element, MG ions shown as green spheres, and IN residues in green are labelled.
